# Letter to the editor: Is the acellular pertussis vaccine driving the increase in severe whooping cough cases in children?

**DOI:** 10.2807/1560-7917.ES.2024.29.37.2400574

**Published:** 2024-09-12

**Authors:** Blandine Prevost, Nadia Nathan, Harriet Corvol

**Affiliations:** 1 Sorbonne University, Paris, France

**Keywords:** Bordetella pertussis, pertussis, whooping cough, children, hospitalisation, asphyxiating cough

**To the Editor:** We read with great interest the report by Rodrigues et al. documenting the resurgence of *Bordetella pertussis*, the main agent of whooping cough, in France in 2024 [1]. Since early 2024, several studies have indeed reported a global increase in whooping cough cases globally, with substantial spikes observed in Europe, Asia and the Americas [2,3]. This resurgence has even resulted in fatalities, notably among infants. In France alone, more than 7,000 cases of pertussis were identified through PCR testing between January and May 2024, in stark contrast to the 500 cases reported for the entire year of 2023 [4]. What is behind this dramatic spike? Could the COVID-19 pandemic have disrupted the usual cycle of whooping cough outbreaks? Is the acellular pertussis vaccine inadvertently facilitating this resurgence?

In the first half of 2024, our paediatric pulmonology unit in France has also seen a marked increase in hospitalisations of children with severe whooping cough, reflecting the broader trends and underscoring the urgency of addressing this public health challenge. Nine previously healthy children (five female and four male children) were hospitalised and diagnosed with *B. pertussis* infection, confirmed through positive PCR results from nasopharyngeal swabs (Table). Among these cases, five were typical presentations: infants younger than 1 year, who had not yet completed their vaccination series, were admitted for episodes of cyanosis or malaise (Table, Cases 1 to 5). However, four cases were notably atypical. Despite being fully vaccinated according to the recommended schedule, these children, aged between 2 and 15 years, were hospitalised with severe symptoms, including asphyxiating coughs, nocturnal respiratory distress and cyanosis (Table, Cases 6 to 9). Case 9 was co-infected with *Mycoplasma pneumonia*, which probably exacerbated the symptoms and led to acute lobar pneumonia. Despite being discharged, these children continued to experience a persistent cough accompanied by episodes of cyanosis at home for several weeks.

**Table:** Medical history of children hospitalised for severe whooping cough, Paris, France, February–August 2024 (n = 9)

Patient	1	2	3	4	5	6	7	8	9
Age group	< 1 year	< 1 year	< 1 year	< 1 year	< 1 year	2–15 years	2–15 years	2–15 years	2–15 years
Pertussis vaccine	None	None	None	Up to date (first dose 1 month ago)	11-month booster not yet administered	Up to date (last booster 18 months ago)	Up to date (last booster 2.5 years ago)	Up to date (last booster 11 months ago)	Up to date (last booster 5 years ago)
Anamnesis									
Household exposure	Yes	Yes	No	Yes	No	No	No	No	No
Symptoms at onset	Cough	Cough	Cough and vomiting	Cough and runny nose	Cough and vomiting	Cough and suffocation	Cough and suffocation	Cough and suffocation	Cough and fever
Cyanosis at home	Yes	Yes	Yes	Yes	Yes	Yes	Yes	Yes	No
Duration of symptoms before admission	1 day	2 days	2 days	5 days	5 days	21 days	2 days	25 days	11 days
Multiplex PCR in nasopharyngeal swabs									
*Bordetella pertussis*	+	+	+	+	+	+	+	+	+
Other	−	−	−	−	Enterovirus	Rhinovirus	−	Rhinovirus	*Mycoplasma pneumoniae*
Outcome									
Admission to ICU	Yes	No	No	No	No	No	No	No	No
Antibiotics	Azithromycin 3 days	Azithromycin 3 days	Azithromycin 3 days	Azithromycin 3 days	Clarithromycin 7 days	Azithromycin 3 days	Azithromycin 3 days	Azithromycin 3 days	Azithromycin 3 days
Respiratory support	NIV: 18 days, oxygen: 9 days	No	Oxygen: 4 days	No	No	No	No	No	Oxygen: 2 days
Duration of hospital stay	43 days	11 days	16 days	3 days	2 days	8 days	2 days	7 days	4 days

+: positive; −: negative; ICU: intensive care unit; NIV: non-invasive ventilation.

This surge is likely to be due to a decline in population immunity after several years of low case numbers. Whooping cough exhibits cyclical outbreaks every 3–5 years. The expected natural increase in cases between 2020 and 2023 was probably suppressed by COVID-19 control measures, delaying the current peak. In addition, the recent low incidence has reduced population immunity, leading to a larger-than-anticipated rise in cases.

Even more concerning are these unusual cases, suggesting the outbreak might involve a strain more resistant to the vaccine, which generally has an 80–85% efficacy rate [5]. This report underscores the importance of re-evaluating current vaccination schedules, particularly those using acellular vaccines in France and globally. Introduced in France in 2013, acellular vaccines offer strong protection but have a shorter duration of immunity compared to earlier whole-cell vaccines [5]. Since this change, an increase in pertussis cases among children aged 2–5 years has been observed since 2017 [6]. The underlying immunological mechanisms contributing to this risk in this age group remain unclear.

Furthermore, recent studies indicate that the introduction of acellular vaccines may have led to an increase in the mutation rate of *B. pertussis* vaccine antigens [7]. This potential evolutionary adaptation may contribute to outbreaks even in well vaccinated populations. The development of acellular vaccines was prompted by concerns about the safety of whole-cell pertussis vaccines. Acellular vaccines, containing purified antigenic components of* B. pertussis*, are indeed associated with fewer adverse events. However, the possibility of vaccine-driven evolution of *B. pertussis* raises important questions about maintaining long-term efficacy and its impact on public health [8].

These observations highlight a need for vigilance and further research to explore whether the acellular nature of the pertussis vaccines might be contributing to a reduction in their duration of effectiveness, which could play a role in the resurgence of severe forms of whooping cough, even in well-vaccinated populations.
